# Ceph-Net: automatic detection of cephalometric landmarks on scanned lateral cephalograms from children and adolescents using an attention-based stacked regression network

**DOI:** 10.1186/s12903-023-03452-7

**Published:** 2023-10-27

**Authors:** Su Yang, Eun Sun Song, Eun Seung Lee, Se-Ryong Kang, Won-Jin Yi, Seung-Pyo Lee

**Affiliations:** 1 Department of Applied Bioengineering, Graduate School of Convergence Science and Technology, Seoul National University, Seoul, South Korea; 2 Department of Oral Anatomy, Dental Research Institute, School of Dentistry, Seoul National University, Seoul, South Korea; 3 Department of Biomedical Radiation Sciences, Graduate School of Convergence Science and Technology, Seoul National University, Seoul, South Korea; 4 Department of Oral and Maxillofacial Radiology and Dental Research Institute, School of Dentistry, Seoul National University, Seoul, South Korea

**Keywords:** Deep learning, Cephalometric landmark, Cephalometric analysis, Landmark detection, Scanned lateral cephalogram

## Abstract

**Background:**

The success of cephalometric analysis depends on the accurate detection of cephalometric landmarks on scanned lateral cephalograms. However, manual cephalometric analysis is time-consuming and can cause inter- and intra-observer variability. The purpose of this study was to automatically detect cephalometric landmarks on scanned lateral cephalograms with low contrast and resolution using an attention-based stacked regression network (Ceph-Net).

**Methods:**

The main body of Ceph-Net compromised stacked fully convolutional networks (FCN) which progressively refined the detection of cephalometric landmarks on each FCN. By embedding dual attention and multi-path convolution modules in Ceph-Net, the network learned local and global context and semantic relationships between cephalometric landmarks. Additionally, the intermediate deep supervision in each FCN further boosted the training stability and the detection performance of cephalometric landmarks.

**Results:**

Ceph-Net showed a superior detection performance in mean radial error and successful detection rate, including accuracy improvements in cephalometric landmark detection located in low-contrast soft tissues compared with other detection networks. Moreover, Ceph-Net presented superior detection performance on the test dataset split by age from 8 to 16 years old.

**Conclusions:**

Ceph-Net demonstrated an automatic and superior detection of cephalometric landmarks by successfully learning local and global context and semantic relationships between cephalometric landmarks in scanned lateral cephalograms with low contrast and resolutions.

## Background

A lateral cephalogram is widely used to analyze face and jaw growth and development to establish malocclusion diagnosis and plan orthodontic treatment such as braces or surgery. This can also provide information regarding the positions of the teeth, face, and jaw to monitor and plan orthodontic treatment [1]. Children and adolescents typically experience skeletal and dental structure changes during development stages. Lateral cephalograms are used to access craniofacial growth and development over time, providing valuable information on the treatment progression and the long-term outcomes of orthodontic treatment.

An essential step in orthodontic treatment planning is cephalometric analysis in a lateral cephalogram, which provides quantitative information regarding the relationship between the dental and skeletal aspects of the human skull according to cephalometric landmarks [2, 3]. The accurate detection of cephalometric landmarks on a lateral cephalogram is important to the success of the cephalometric analysis [4]. The quantitative evaluation of the angles and distances between cephalometric landmarks provides anatomical information and surrounding soft-tissue aberrations and helps in evaluating the craniofacial growth pattern. Image quality is a primary consideration in cephalometric landmark detection, and during the conversion of analog cephalometric radiographs to digital format, the quality of the original film is a major factor that affects landmark identification [5].

Analog cephalometric radiographs of poor quality can appear worse on screen and can lead to greater errors in digital technology [6]. Furthermore, manual cephalometric analysis is time-consuming and can cause inter- and intra-observer variability [7, 8]. Also, when conducting large data analysis, even experienced researchers get stuck on maintaining accuracy and consistency [9]. Therefore, automatic methods are required to detect cephalometric landmarks for orthodontic diagnosis and treatment planning.

For many years, analog and scanned radiographs were the standard in the medical field. Recently, advances in digital technologies have transformed the field of radiography, making it possible to obtain high-quality digital radiographs that can be used to diagnose and treat a wide range of medical conditions [10, 11]. Digital radiographs offer advantages such as high resolution and convenience, and they are now the preferred method of imaging in most medical fields. The main difference between scanned and digital radiographs is the method of image acquisition: scanned radiographs are obtained by scanning analog film with a film scanner, while digital radiographs are captured directly in a digital format by a digital X-ray detector. Digital radiographs offer the optimal combination of image quality, cost, portability, and ease of manipulation. However, they may not be available in all environments. Analog and scanned radiographs are still used in some settings, such as developing countries and medically underserved areas, where digital infrastructure is not available. In developing countries and medically underserved areas, there may be a shortage of skilled radiologists to interpret scanned lateral cephalograms. An automatic method for cephalometric landmark detection can help to improve the accuracy and consistency of cephalometric analysis in scanned lateral cephalograms and reduce the time-consuming and labor-intensive processes.

Automatic landmark detection on scanned lateral cephalograms from children and adolescents remains challenging due to three major reasons. First, there are morphological variations in anatomy and growth among different children and adolescents, which lead to significant variations in anatomical landmarks [12]. These morphological variations are caused by differences in anatomical size and shape including supernumerary teeth, primary teeth, unerupted teeth, and permanent teeth. Second, children and adolescents have a lower bone density than adults, which can result in image radiolucency in lateral cephalograms. In these radiolucent images, cephalometric landmarks may not always be identified, particularly if they are located in areas where there are several overlaps with other anatomical structures [13]. Lastly, scanned lateral cephalograms have lower image quality than digital lateral cephalograms. Scanned lateral cephalograms relatively have low contrast and resolutions in the anatomical structures, making it can be difficult to accurately identify cephalometric landmarks [14, 15].

In recent years, deep learning-based methods for cephalometric landmark detection outperformed other conventional image processing and machine-learning approaches [2, 16–18]. Also, remarkable success was achieved using a fully convolutional network (FCN) [19–22]. Lee et al. proposed an end-to-end deep learning method for cephalometric landmark detection in digital lateral cephalograms using a public dataset [23]. The experimental results showed superior performance by successfully localizing the cephalometric landmarks within significant margins from the ground truths. Oh et al. proposed a novel CNN framework for cephalometric landmark detection on a public dataset to learn deep anatomical context features using an anatomical perturbation approach [24]. Zeng et al. reported a cascaded three-stage CNN framework to detect cephalometric landmarks in digital lateral cephalograms accurately [25]. Jiang et al. proposed transformer-based two-stage networks which learned the correlations between local–global anatomical features in a coarse-to-fine manner for cephalometric landmark detection [26]. Furthermore, several previous approaches typically follow two-stage deep networks [16–18]. In the first stage, for region proposals or extracting regions of interest (ROI), coarse candidates of landmarks are identified. In the second stage, referred to as the refinement stage, ROIs extracted in the first stage are passed through another deep network that performs fine-grained detection of a fine coordinate of a specific landmark in the region proposals. However, such methods are dependent on the accuracy of the first stage and hence are far from an end-to-end training manner. Furthermore, since forward execution is independently required for each region proposal, it is very time-consuming and computationally expensive. Although existing methods attained significant progress, joint learning of the anatomical contextual features such as local and global relationships of cephalometric landmarks during training is lacking and therefore is a limitation, leading to a suboptimal result. In addition, most existing studies have reported automatic detection methods for cephalometric landmarks in digital lateral cephalograms, while as far as we know that no studies have been reported in scanned lateral cephalograms.

The purpose of this study was to automatically detect cephalometric landmarks on scanned lateral cephalograms with low contrast and resolution using an attention-based stacked regression network (Ceph-Net). Ceph-Net was an end-to-end encoder-decoder architecture, which tied three two-dimensional (2D) FCNs including multi-scale inputs (MSI), a dual attention module (DSAM), a multi-path convolution module (MCM), and deep supervision. Ceph-Net was evaluated on a test dataset which consists of 400 scanned lateral cephalograms obtained over 8 years from 50 patients aged 8 to 16 except for 12 years old. We compared the detection performance of Ceph-Net with those of popular detection networks including U-Net [27], SegNet [28], Dense U-Net [29], and Attention U-Net [30]. Our main contributions are as follows: (1) We proposed an attention-based stacked regression network that improved the high-resolution representation through dense stacking of three FCNs to learn fine-grained details of cephalometric landmarks in a 2D heatmap. (2) We used DSAM to capture local and global context and semantic relationships between cephalometric landmarks in scanned lateral cephalograms. (3) We employed categorical cross-entropy loss (CEL) with intermediate supervision in Ceph-Net to further improve the detection performance, which promoted more direct backpropagation to convolutional layers for a faster convergence and better detection accuracy.

## Methods

### Data acquisition and preparation

In this study, a total of 1286 scanned lateral cephalograms were used from 267 patients (mean age: 11.9 years; age range: 8–16 years; 129 females, 138 males) who underwent lateral cephalography (Seoul National University, School of Dentistry, Republic of Korea) for oral health status and diagnosis of oral diseases between 1995 and 2003. In 267 patients followed for 8 years, 502 images were intermittently obtained from 169 patients, while 784 images were obtained annually from 98 patients, one each year. Ethical approval (S-D20210028) for this study was obtained from the research ethics committee of Seoul National University, School of Dentistry, which waived the requirement for informed consent from all participants due to the nature of the retrospective study. All experiments were conducted in accordance with the approved guidelines.

**Fig. 1:**
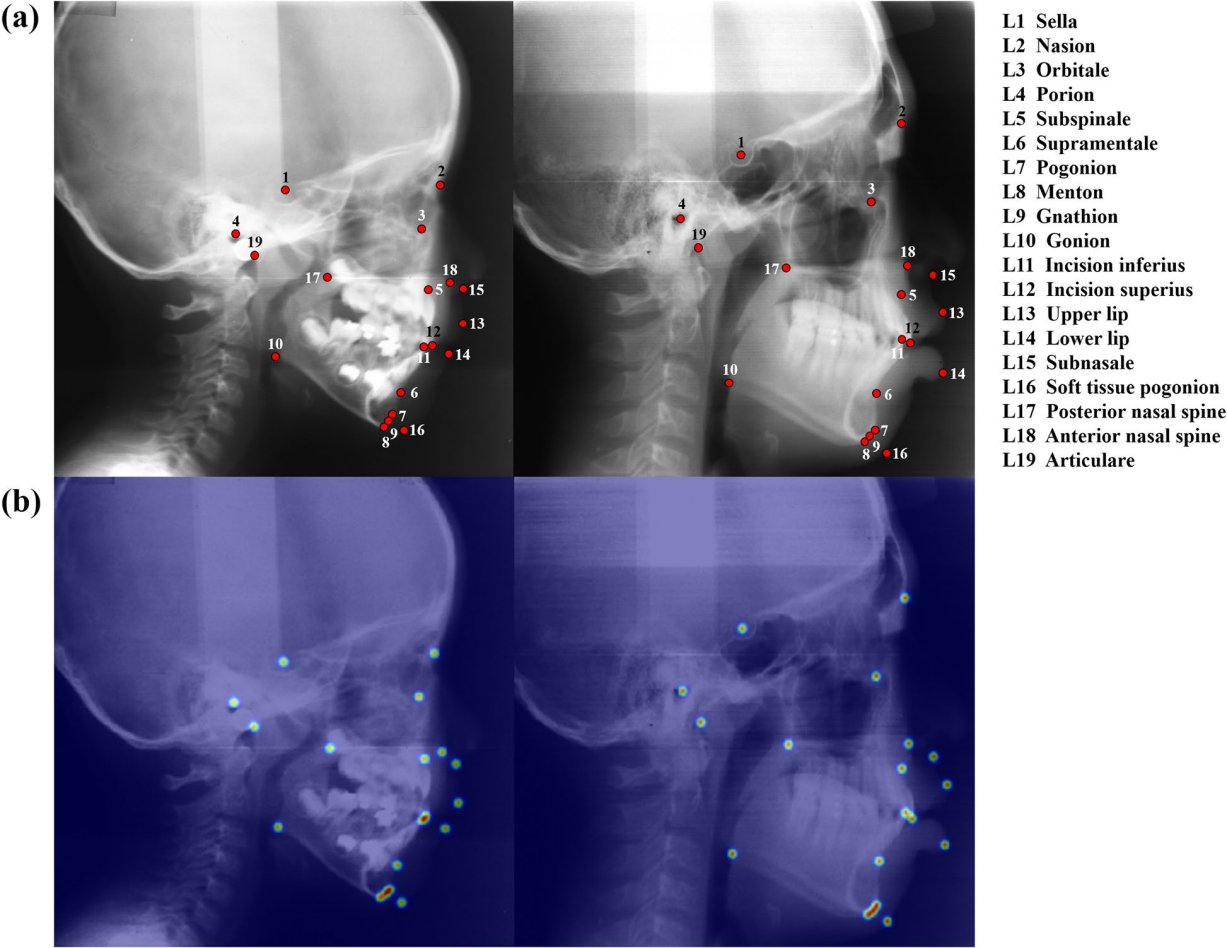
**a** Examples of scanned lateral cephalograms with labeling of 19 cephalometric landmarks. **b** 2D heatmap generations from manual labeling results

An experienced oral and maxillofacial radiologist manually annotated 19 cephalometric landmarks on a scanned lateral cephalogram using Labelbox (Labelbox Inc., San Francisco, California, USA). As shown in Fig. 1, the cephalometric landmarks include the sella, nasion, orbitale, porion, subspinale, supramentale, pogonion, menton, gnathion, gonion, incision inferius, incision superius, upper lip, lower lip, subnasale, soft-tissue pogonion, posterior nasal spine, anterior nasal spine, and articulare [31]. We performed inter-observer validation by two radiologists, one with about 10 years of clinical experience and the other with about 5 years of clinical experience. The mean inter-observer variability of the two radiologists was 1.51 ± 3.94 mm on the test set. The number of scanned lateral cephalograms in training, validation, and test datasets were split into 704, 182, and 400 images, respectively. Analog cephalometric radiographs were scanned by using a film scanner (Epson Perfection V850 Pro, Seiko Epson Corp., Tokyo, Japan) at 300 dpi and exported as images of TIF format. Scanned lateral cephalograms (2400 × 3000) were resized to a size of 576 × 736 pixels which was based on the size used in a previous study [25]. Image calibration was performed using manual measurement and ImageJ software (National Institute of Health, Bethesda, Maryland, USA). The manual measurement was performed using a ruler for an analog cephalometric radiograph and ImageJ for a scanned lateral cephalogram, with 10 mm as the reference length [14, 32, 33]. Measurement of the reference length was converted from mm to pixels using ImageJ, where a calibration ratio of each pixel was equal to 0.1 mm on 2400 × 3000 pixels.

### 2D heatmap generation

We adopted a heatmap-based landmark detection method that transfers cephalometric landmark coordinates into a 2D Gaussian heatmap [20]. A set of the *x* and *y* coordinates of the cephalometric landmark $L\in {\mathbb{R}}^{i\times 2}$ is represented by a separate 2D Gaussian heatmap $H(x;{L}_{i},{\sigma }_{i})\in {\mathbb{R}}^{H\times W}$ corresponding to a scanned lateral cephalogram $I\in {\mathbb{R}}^{H\times W}$, where $i$ is the number of cephalometric landmarks. Each pixel value in a heatmap $H$ is regarded as a probability of the cephalometric landmark in the range of 0 to 1. The probability value of a pixel is $1.0$ at the center of a 2D heatmap, and the probability values decrease further away from the center. ${L}_{i}$ indicates a cephalometric landmark, and $H(x;{L}_{i},{\sigma }_{i})$ is defined as the Gaussian function:

1 $$H(x,y;{L}_{i,}{\sigma }_{i})=\frac{\varnothing }{\sigma \sqrt{2\pi }}\mathrm{exp}\left[-\frac{1}{2{\sigma }_{i}^{2}}\left({\left(x-{L}_{i}^{x}\right)}^{2}+{\left(y-{L}_{i}^{y}\right)}^{2}\right)\right]$$

where ${L}_{i}^{x}$ and ${L}_{i}^{y}$ are the* x* and *y* coordinates of the cephalometric landmark ${L}_{i}$, while *i* is the range of 1 to 19. The $\sigma$ is a standard deviation which is the hyperparameter that determines the sharpness of the 2D Gaussian distribution. $\varnothing$ is the scale factor to define the region size of a 2D heatmap, empirically set as 5. A heatmap pixel $x$ with a lower $\sigma$ shows a much sharper distribution than a higher $\sigma$ in centers of landmarks, leading to sensitive cephalometric landmark detection. We used scanned lateral cephalograms as input images and the 20-channel heatmaps $H$ of cephalometric landmarks as ground truth for training (Fig. 1b).

### Overall procedures of the proposed method

**Fig. 2:**
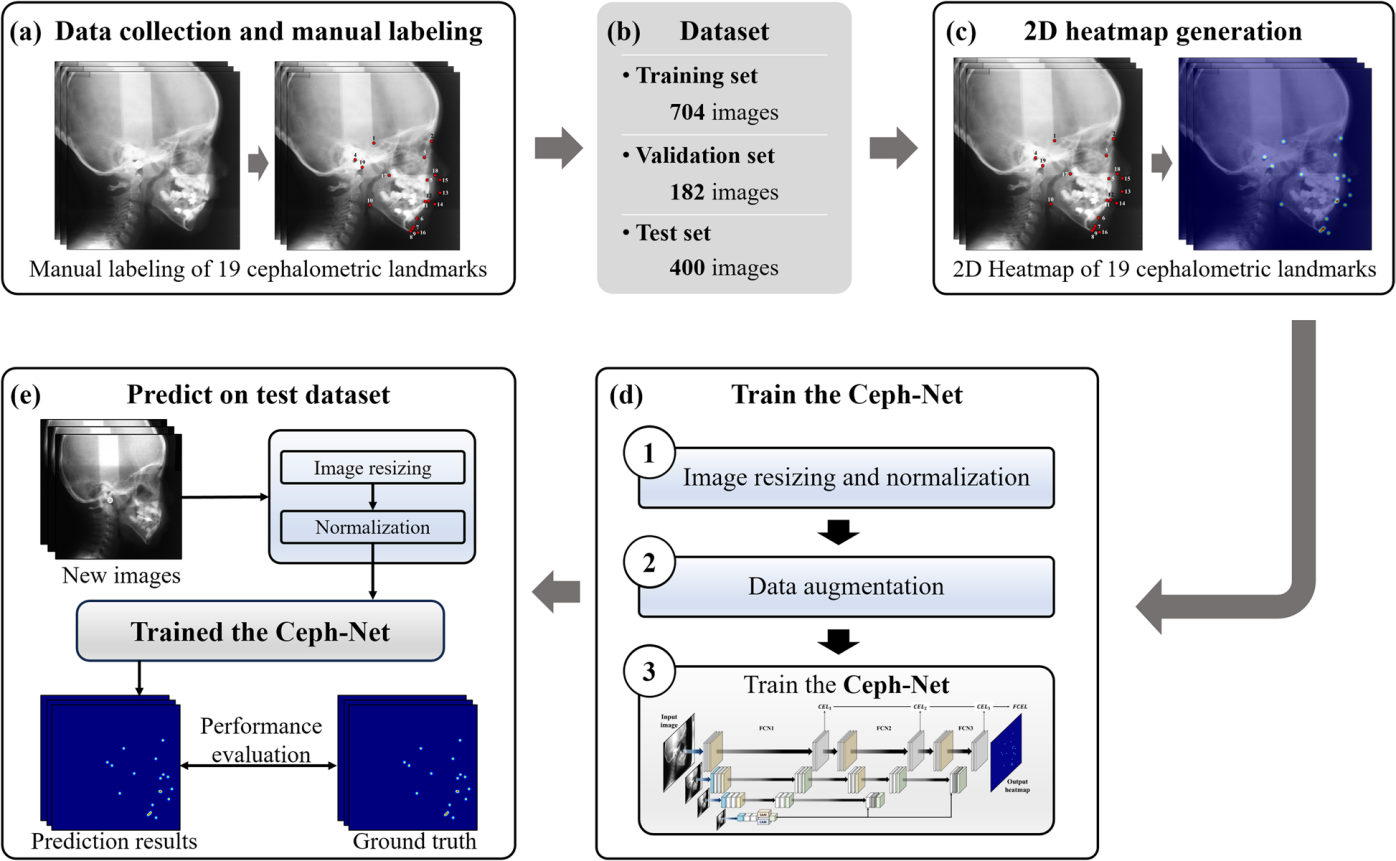
The schematic diagram of the proposed method. **a** Data collection and manual labeling of cephalometric landmarks. **b** Dataset composition. **c** 2D heatmap generation from manual labeling results. **d** The training process of the Ceph-Net. **e** The prediction and evaluation process of the Ceph-Net

In this study, the entire process of our proposed method was divided into five procedures (Fig. 2). The first step is data collection and manual labeling of 19 cephalometric landmarks in scanned lateral cephalograms. The second is data composition for dividing training, validation, and test dataset. The third is the 2D heatmap generation from manually labeled 19 cephalometric landmarks to train the Ceph-Net based on a heatmap-based landmark detection approach. The fourth is the training process of the Ceph-Net including image resizing and normalization, data augmentation, and training of the Ceph-Net. The last is the prediction and evaluation process of the Ceph-Net. The Ceph-Net automatically detected 19 cephalometric landmarks from a scanned lateral cephalogram in an end-to-end manner.

### Attention-based stacked regression network (Ceph-Net)

**Fig. 3:**
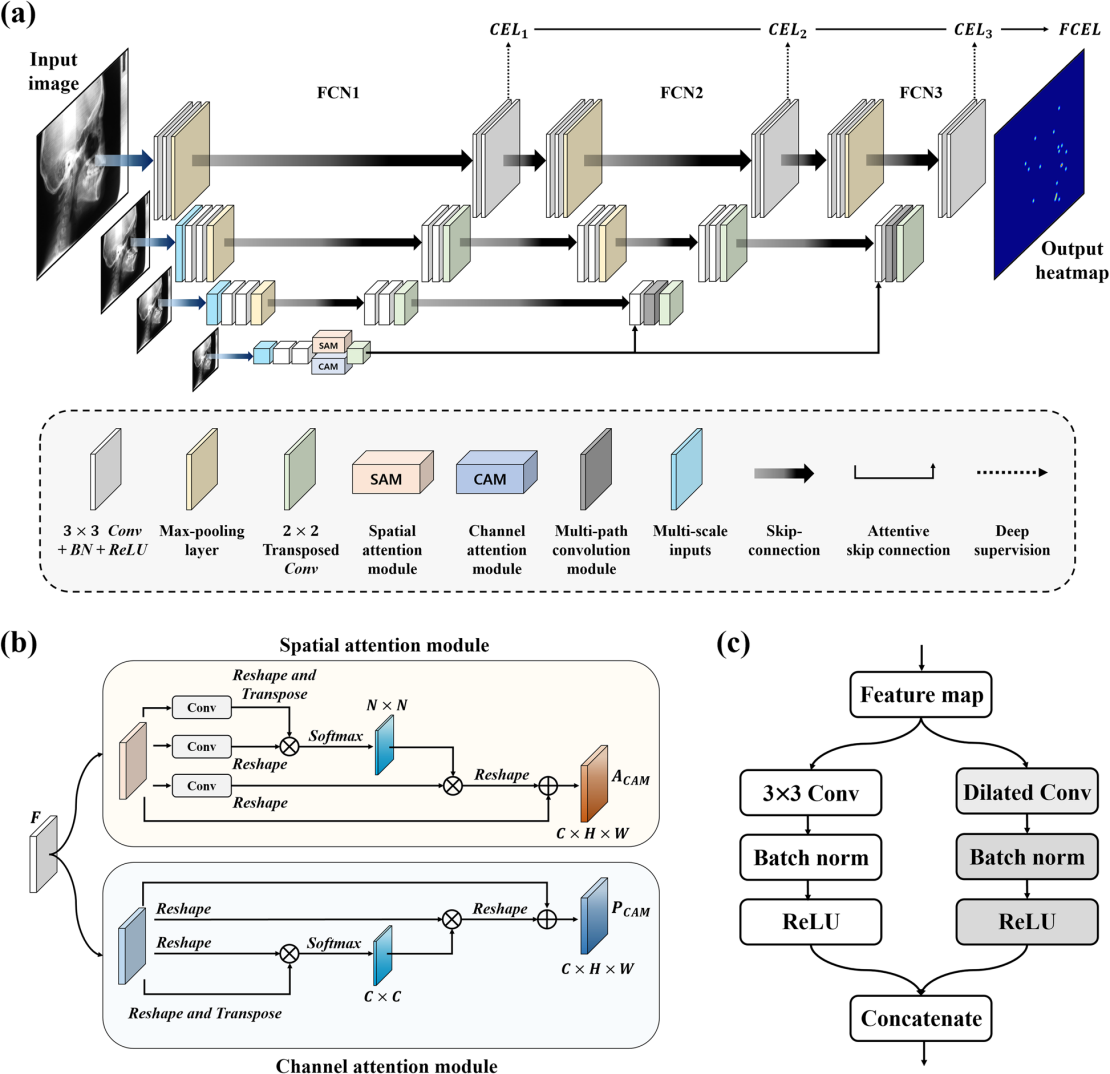
**a** The network architecture of the proposed Ceph-Net. The schematics of (**b**) and (**c**) are the dual attention module and multi-path convolution module, respectively

In this study, we proposed an attention-based stacked regression network named Ceph-Net that directly regressed a 2D heatmap from an input image for cephalometric landmark detection. As shown in Fig. 3a, Ceph-Net was an end-to-end encoder-decoder architecture, which tied three FCNs including MSI, DSAM, MCM, and deep supervision. The encoder-decoder architecture consisted of 2D convolution blocks including a $3\times 3$ convolutional layer, batch normalization (BN), and rectified linear unit (ReLU) activation except the output layer. The max-pooling and transposed convolutional layers with a stride of 2 were used for down- and up-sampling, respectively. Skip-connections were employed between an encoder and a decoder. According to the depth of the FCNs, the number of feature maps gradually increased from 16 to 32, 64, and 128 in encoder parts, while they gradually decreased from 128 to 64, 32, and 16 in decoder parts. To mitigate spatial information loss, MSI was used at each level of the encoding layer in the first FCN and generated by multiplying $2\times 2$, $4\times 4$, and $8\times 8$ average pooling operations with an input image. Then, feature maps from resized inputs were acquired by a 2D convolution block and concatenated with a down-sampled feature map at each level of the encoding layer, the number of feature maps was the same as those at each level of encoding layer. The last output layer of Ceph-Net was a $3\times 3$ convolutional layer with a *Softmax* activation function.

In automatic landmark detection tasks, landmarks require different semantics due to variations in the shape and size of anatomical structures among patients. Attention mechanisms in deep learning were inspired by the human visual cognition system, which could encourage deep networks to more focus on the relevant areas and ignore the background by weighting to different areas in an image [30, 34, 35]. Also, attention mechanisms were widely used to capture complex semantic relationships in medical image analysis [36]. Based on this observation, we used DSAM to integrate local features with their corresponding global relationships of cephalometric landmarks [34]. The DSAM consisting of the spatial attention module (SAM) and the channel attention module (CAM) was embedded in the bridge of the first FCN as shown in Fig. 3b.

The SAM captures long-range spatial relationships in original feature maps. To extract the spatial attention map, the original feature map $F\in {\mathbb{R}}^{C\times H\times W}$ is fed to SAM, where $C, W,$ and $H$ indicate the channel, width, and height dimensions, respectively. Specifically, new feature maps ${F}_{0}$ and ${F}_{1}\in {\mathbb{R}}^{C\times H\times W}$ are generated by a convolutional layer. Then, ${F}_{0}$ is reshaped to ${\mathbb{R}}^{C\times N}$, and ${F}_{1}$ is transposed to ${\mathbb{R}}^{C\times N}$, where $N$ represents $H\times W$. We performed a matrix multiplication between ${F}_{0}$ and ${F}_{1}$ and applied a softmax activation to generate the spatial attention map $P\in {\mathbb{R}}^{N\times N}$:

2 $${p}_{i,j}=\frac{\mathrm{exp}({F}_{0,i}\otimes {F}_{1,j})}{{\sum }_{i=1}^{N}\mathrm{exp}({F}_{0,i}\otimes {F}_{1,j})}$$

where ${p}_{i,j}$ measures the impact of the ${i}^{th}$ position on the ${j}^{th}$ position. The original feature map $F$ is fed into a different convolutional layer to extract ${F}_{2}\in {\mathbb{R}}^{C\times H\times W}.$ The ${F}_{2}$ is reshaped to ${\mathbb{R}}^{C\times N}$. Thereafter, a matrix multiplication between ${F}_{2}$ and $P$ transposition was performed, and the results were reshaped to ${\mathbb{R}}^{C\times H\times W}$. The final spatial attention feature map ${P}_{SAN}$ is obtained as:

3 $${P}_{SAM,j}={\gamma }_{s}\sum_{i=1}^{N}{p}_{i,j}{F}_{2,i}+{F}_{j}$$

where ${\gamma }_{s}$ is a scale factor set as 0 and gradually learned to assign more weight to the spatial feature map. The SAM aggregates weighted features of all positions into the original features, capturing global context information in feature maps. To selectively highlight important features and suppress unnecessary ones, CAM captures inter-dependencies among channels. The channel attention map $A\in {\mathbb{R}}^{C\times C}$ is directly calculated from the original features $F\in {\mathbb{R}}^{C\times H\times W}$ by the CAM. Specifically, the $F$ is reshaped and transposed in the first branches of the CAM, leading to the ${F}_{0}\in {\mathbb{R}}^{C\times N}$ and the ${F}_{1}\in {\mathbb{R}}^{N\times C}$. A matrix multiplication was performed between ${F}_{0}$ and ${F}_{1}$, and a softmax activation to extract the channel attention map $A\in {\mathbb{R}}^{C\times C}$ was then applied:

4 $${a}_{i,j}=\frac{\mathrm{exp}({F}_{0,i}\otimes {F}_{1,j})}{{\sum }_{i=1}^{C}\mathrm{exp}({F}_{0,i}\otimes {F}_{1,j})}$$

where ${a}_{i,j}$ measures the impact of the ${i}^{th}$ channel on the ${j}^{th}$. We multiply $A$ with the transpositions of $F$, that is, ${F}_{2}$, then reshape the results to ${\mathbb{R}}^{C\times H\times W}$. The final channel attention feature map is obtained as:

5 $${A}_{CAM,j}={\gamma }_{c}\sum_{i=1}^{C}{a}_{i,j}{F}_{2,i}+{F}_{j}$$

where ${\gamma }_{c}$ is a scale factor initially set as 0 and gradually learned. The CAM aggregates weighted features of all the channels into the original features, capturing long-range semantic relationships, and improving feature discriminability between classes. In Ceph-Net, the spatial and channel attention feature maps were extracted in the bridge of the FCN1 using the DSAM and concatenated at the next bridges of FCNs with up-sampling through attentive skip-connections. Furthermore, we introduced MCM consisting of two parallel convolution paths to capture features with different scales of receptive fields (Fig. 3c). The MCM input was the combined feature maps from the attentive and skip-connections. In the MCM, the left convolution path consisted of a $3\times 3$ convolutional layer, BN, and ReLU, while a dilated convolutional layer was adopted to enhance receptive fields at the right convolution path. After capturing features by MCM with different scales of receptive fields, the concatenated feature maps were fed to the decoder. The MCMs with dilated rates of 2 and 3 were used at FCN2 and FCN3, respectively.

We used popular detection networks including U-Net [27], SegNet [28], Dense U-Net [29], and Attention U-Net [30] to compare the detection performance of cephalometric landmarks with Ceph-Net. U-Net [27] is one of the popular deep networks for medical image analysis. It consisted of an encoder path with five levels to capture context and a symmetric decoder path to recover image resolution to those of inputs. U-Net had approximately 7.7 million trainable parameters. SegNet [28] had a deep encoder-decoder architecture for semantic pixel-wise detection. The encoder had 13 convolution layers with BN and a max-pooling layer of stride 2. The decoder had the same number of convolution layers and performed the up-sampling using the un-pooling layer. SegNet had approximately 29.4 million trainable parameters. Dense U-Net [29] had a U-shape structure similar to U-Net, where densely connected blocks [29] were used in the encoder path for efficient feature extraction. Dense U-Net had approximately 15.4 million trainable parameters. Attention U-Net [30] was a novel attention network for medical image analysis. The attention module was used in the decoder part to focus on target structures of varying sizes and shapes. The attention module could be integrated into standard CNN architectures with minimal computational cost while increasing the deep network sensitivity and accuracy. Attention U-Net had approximately 7.9 million trainable parameters.

### Loss function with deep supervision

For network training, we employed CEL to measure the difference between the true probability distribution and the predicted probability distribution [37]. CEL is used to train deep networks by minimizing the difference between the predicted and true probability distributions during the backpropagation step. CEL is defined as:

6 $$CEL\left(y,\widehat y\right)=-\frac1N\sum_{i=1}^N(y_i\cdot\log\widehat{y_i}+\left(1-y_i\right)\cdot\log(1-\widehat{y_i}))$$

where $y$ and $\widehat{y}$ are ground truth and prediction results, respectively. $N$ is the sample size. The CEL with deep supervision (FCEL) is then defined as a sum of a loss from intermediate deep supervision and defined as:

7 $$FCEL\left(\mathrm{y},\widehat{y}\right)= {CEL}_{1}\left(y,{\widehat{y}}_{1}\right)+{CEL}_{2}\left(y,{\widehat{y}}_{2}\right)+{CEL}_{3}\left(y,{\widehat{y}}_{3}\right)$$

where $y$ and $\widehat{y}$ are ground truth and prediction from intermediate deep supervision at each FCN. In Ceph-Net, the FCEL improved training stability and detection accuracy for cephalometric landmarks.

### Training setup

The detection networks were trained using the RMSprop optimizer for 100 epochs with an initial learning rate of 10^–4^, which decreased by a factor of 0.5 when the validation loss stopped decreasing for 25 epochs. A batch size of 8 and a single GPU with 24 GB RAM were used. All detection networks were implemented in Python3 using the Keras framework with the TensorFlow backend. The data augmentation procedure consisted of geometry and intensity transformation including random rotation (− 10–10 degrees), zoom (0.95–1.05), and intensity changes (− 50%–50%).

### Evaluation metrics

Ceph-Net was evaluated on a test dataset which consisted of 400 scanned lateral cephalograms obtained over 8 years from 50 patients aged 8 to 16 except for 12 years old. The detection performance for the 19 cephalometric landmarks was evaluated using the mean radial error (MRE) and the successful detection rate (SDR) [31]. To extract coordinates of predictive cephalometric landmarks, maximum responses in predicted 2D heatmaps were obtained from detection networks. The MRE is defined as:

8 $$\mathrm{MRE}=\frac{1}{N}{\sum }_{i=1}^{N}{R}_{i}$$

where $n$ indicates the number of samples and $R$ indicates the Euclidean distance between ground truth and a predictive result. The SDR shows the percentage of successfully detected landmarks in the range of 1.0, 2.0, 3.0, 4.0, and 5.0 mm errors.

Seven standard clinical measurements for classifications [25, 38–40] of anatomical types were used to compare the accuracy of cephalometric analysis (Table 3) [16, 17, 24, 25]. Seven standard clinical measurements included (1) ANB: The angle between subspinale, nasion, and supramentale; (2) SNB: The angle between sella, nasion, and supramentale; (3) SNA: The angle between sella, and nasion, subspinale; (4) ODI (Overbite depth indicator): Sum of the angle between the lines from subspinale to supramentale (AB plane) and from menton to gonion (Mandibular plane), and the angle between the lines from the posterior nasal spine to the anterior nasal spine (Palatal plane) and from porion to orbitale (Frankfort horizontal plane); (5) APDI (Anteroposterior dysplasia indicator): Sum of the angle between the lines from porion to orbitale (FH plane) and from nasion to pogonion (Facial Plane), the angle between the lines from nasion to pogonion (FP plane) and from subspinale to supramentale (AB plane), and the angle between the lines from porion to orbitale (FH plane) and from the posterior nasal spine to the anterior nasal spine(Palatal plane); (6) FHI (Facial height index): Ratio of the posterior face height (distance from sella to gonion) to the anterior face height (distance from nasion to menton); (7) FMA (Frankfort mandibular angle): Angle between the lines from sella to nasion and from gonion to gnathion [31, 41–43]. The ground truth and classification results by Ceph-Net for anatomical types (Class 1–3) of seven standard clinical measurements were determined by each angle of them according to Table 4. Classification accuracy of anatomical types is defined as:

9 $$\mathrm{Accuracy}=\frac{Number\;of\;correct\;classifications}{Total\;number\;of\;classifications}\times 100$$

where the correct classification means the classification result produced by Ceph-Net matches the ground truth.

## Results

**Fig. 4:**
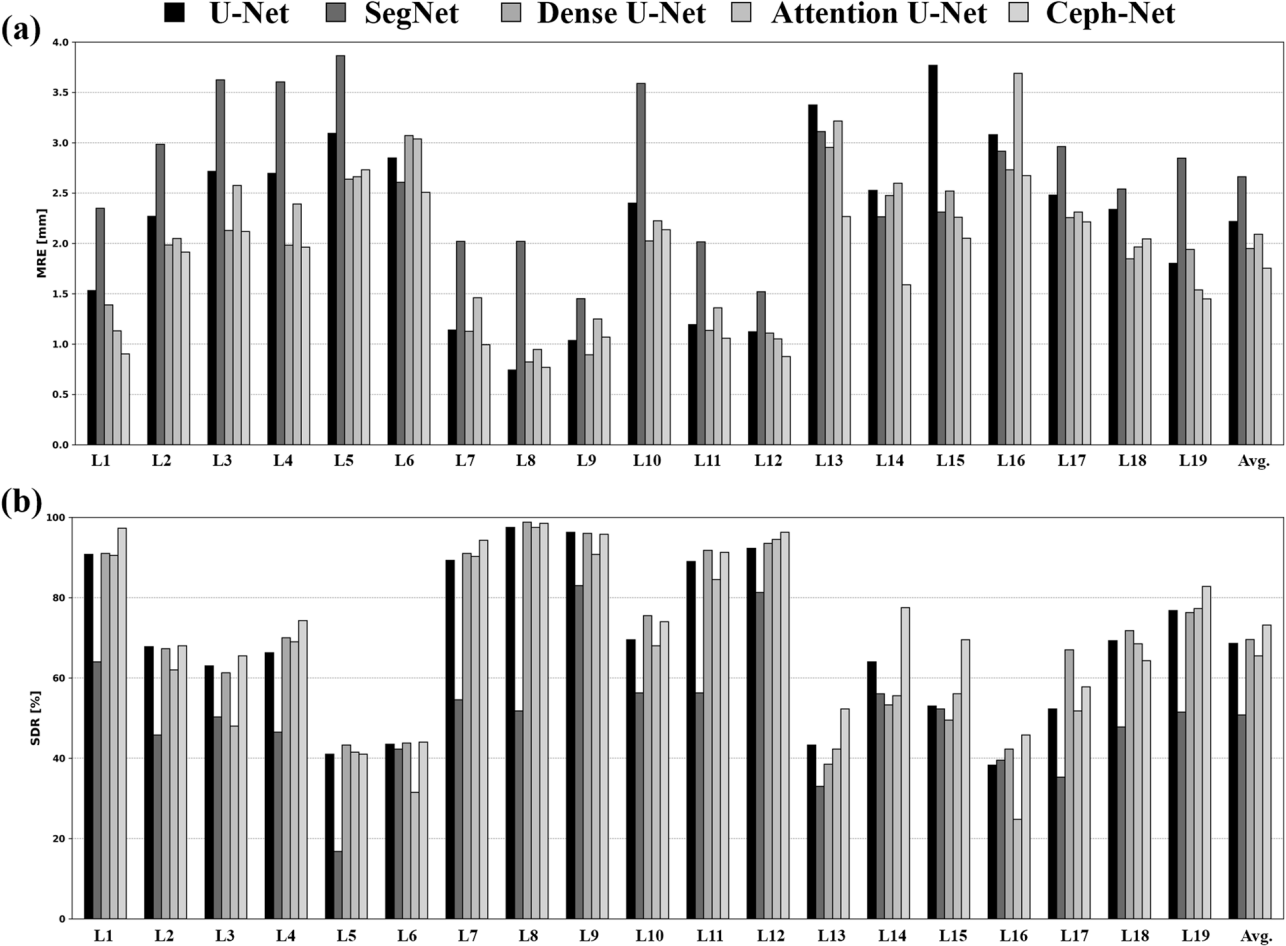
Bar plots for detection performance of cephalometric landmarks from different detection networks. **a** presents the mean radial error of each cephalometric landmark from different detection networks. **b** presents the successful detection rate (less than 2.0 mm errors) of each cephalometric landmark from different detection networks. The abbreviation of each cephalometric landmark is shown in Fig. 1

**Fig. 5:**
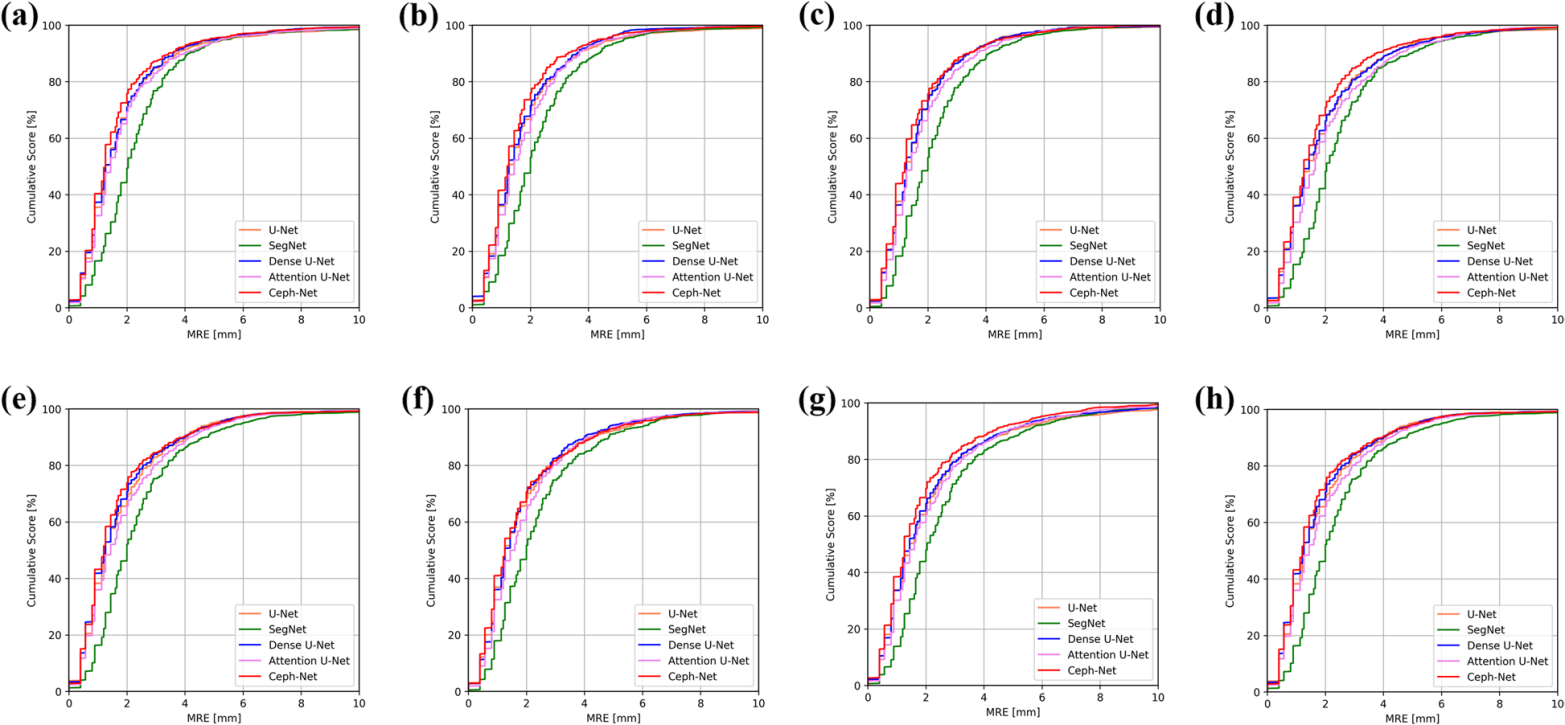
**a**-**h** Show cumulative curves of MREs by different detection networks tested on patients aged 8 to 16 years old, excluding 12 years old sequentially. The orange, green, blue, pink, and red lines indicate cumulative MREs of U-Net, SegNet, Dense U-Net, Attention U-Net, and Ceph-Net, respectively

The landmark detection performance of Ceph-Net was compared with those of popular detection networks such as U-Net [27], SegNet [28], Dense U-Net [29], and Attention U-Net [30]. Table 1 shows the quantitative results of the detection performance of cephalometric landmarks by different detection networks, where our Ceph-Net outperforms the popular detection networks by obtaining the MRE of $1.75\pm 1.67$ mm, and the SDR of $41.35\mathrm{\%}, 73.14\mathrm{\%}, 85.22\mathrm{\%}, 91.18\mathrm{\%},\mathrm{ and }94.65\mathrm{\%}$ in the range of 1.0, 2.0, 3.0, 4.0, and 5.0 mm errors, respectively. Ceph-Net demonstrated the detection performance of MRE under 2.0 mm in detecting sella, nasion, porion, pogonion, menton, gnathion, incision inferius, incision superius, lower lip, and articulare (Table 2). The results showed the detection performance for each of the 19 cephalometric landmarks obtained by different detection networks (Fig. 4). Compared with U-Net, SegNet, Dense U-Net, and Attention U-Net, Ceph-Net achieved lower MRE in detecting these 14 cephalometric landmarks located at the hard tissue (e.g., sella, nasion, orbitale, porion, supramentale, pogonion, incision inferius, incision superius, posterior nasal spine, and articulare) and the soft tissue (e.g., upper lip, lower lip, subnasale, and soft-tissue pogonion). We compared the performance of cephalometric landmarks by different detection networks on the test dataset split by each age (8 to 16 except for 12 years old) as shown in Fig. 5. The cumulative curves of MREs obtained by different detection networks, where Ceph-Net presented the highest detection rate and consistent accuracy compared to popular detection networks (Fig. 5).

**Table 1:** Quantitative comparisons of landmark detection performance with different detection networks using successful detection rate (SDR) and mean radial error (MRE)

Models	SDR (%)					MRE (mm)
	1.0	2.0	3.0	4.0	5.0	
U-Net	36.40	68.56	83.15	90.10	93.90	2.21 ± 6.62
SegNet	16.78	50.72	74.75	86.46	91.88	2.66 ± 4.50
Dense U-Net	36.85	69.55	83.18	90.60	94.51	1.94 ± 3.64
Attention U-Net	32.57	65.47	80.89	89.17	93.67	2.08 ± 3.17
Ceph-Net	41.35	73.14	85.22	91.18	94.65	1.75 ± 1.67

**Table 2:** The detection performance of each cephalometric landmark in Ceph-Net using successful detection rate (SDR) and mean radial error (MRE) with standard deviation (SD)

Landmarks	SDR (%)					MRE (mm)
	1.0	2.0	3.0	4.0	5.0	
Sella	70.25	97.25	98.50	98.75	99.00	0.90 ± 0.83
Nasion	34.50	68.00	84.75	91.25	93.25	1.91 ± 1.74
Orbitale	31.25	65.50	79.25	85.75	91.00	2.11 ± 1.80
Porion	39.25	74.25	87.00	90.50	92.75	1.96 ± 2.21
Subspinale	15.75	41.00	65.25	81.50	89.00	2.73 ± 1.82
Supramentale	16.00	44.00	68.75	82.50	92.75	2.50 ± 1.52
Pogonion	59.00	94.25	99.25	99.75	99.75	0.99 ± 0.66
Menton	77.75	98.50	99.75	99.75	99.75	0.76 ± 0.49
Gnathion	53.50	95.75	99.50	99.75	99.75	1.06 ± 0.54
Gonion	41.50	74.00	80.75	84.50	87.00	2.13 ± 2.47
Incision inferius	64.00	91.25	95.75	97.75	98.75	1.05 ± 0.95
Incision superius	72.25	96.25	98.75	98.75	99.00	0.87 ± 0.86
Upper lip	22.25	52.25	75.25	88.75	95.00	2.26 ± 1.72
Lower lip	42.75	77.50	92.00	95.25	96.25	1.58 ± 1.51
Subnasale	31.50	69.50	81.75	87.75	92.25	2.04 ± 1.90
Soft tissue pogonion	19.50	45.75	63.50	79.50	89.25	2.67 ± 1.80
Posterior nasal spine	23.50	57.75	76.00	87.25	93.00	2.21 ± 1.69
Anterior nasal spine	24.50	64.25	81.75	88.00	94.00	2.04 ± 1.55
Articulare	46.75	82.75	91.75	95.50	97.00	1.44 ± 1.32
Mean (SD)	41.35	73.14	85.22	91.18	94.65	1.75 ± 1.67

**Fig. 6:**
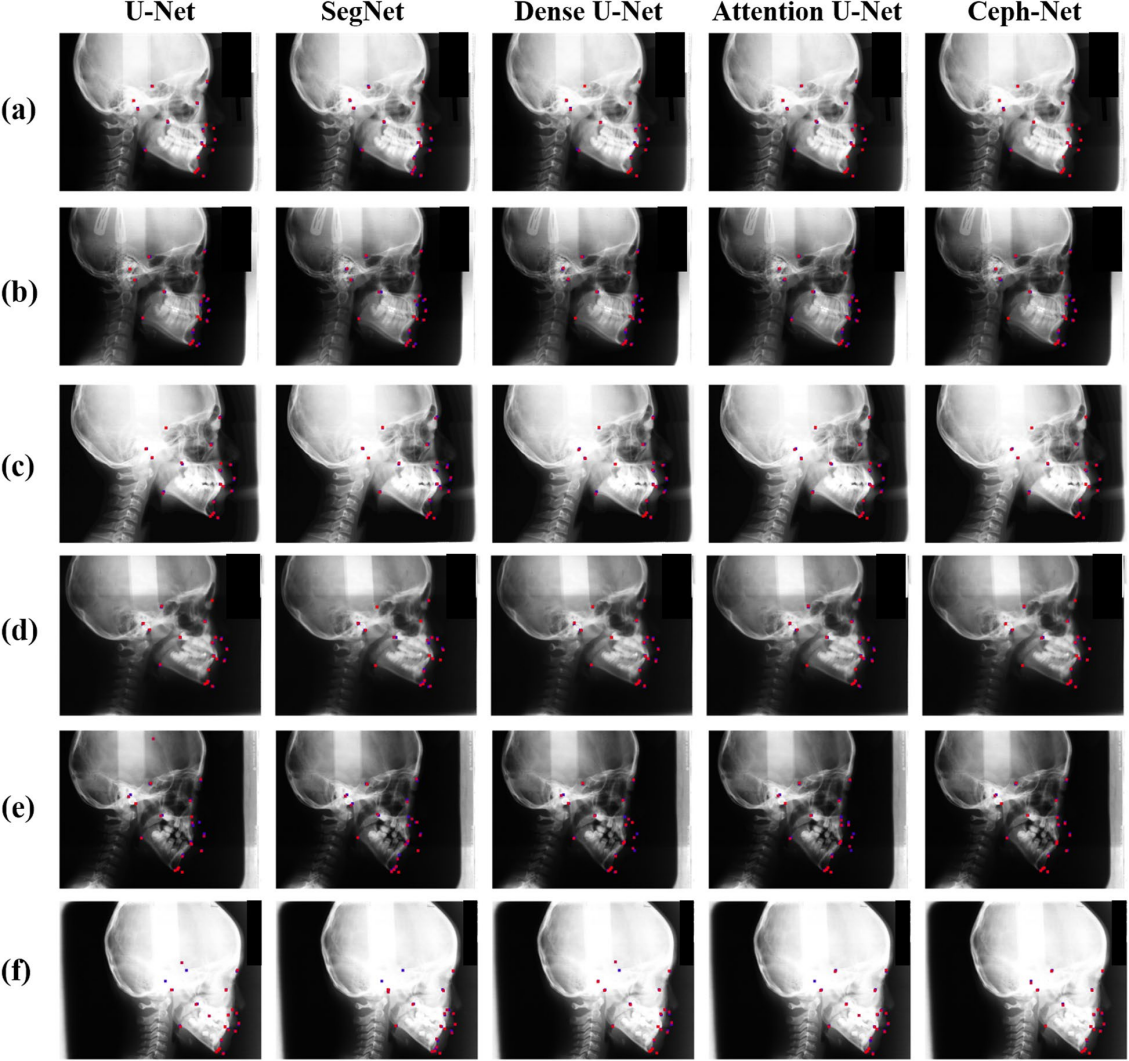
**a**-**f** Show representative detection results of cephalometric landmarks from different detection networks. The red points denote the detected landmarks by detection networks, while the blue points indicate the ground truth of cephalometric landmarks

**Fig. 7:**
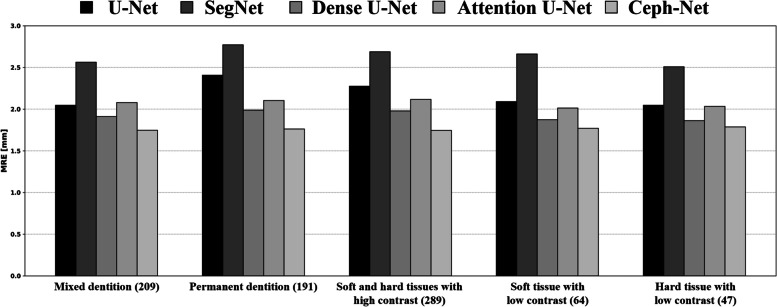
Bar plot for detection performance of cephalometric landmarks from different detection networks on five specific conditions in scanned lateral cephalograms. The bracket means the number of samples

**Fig. 8:**
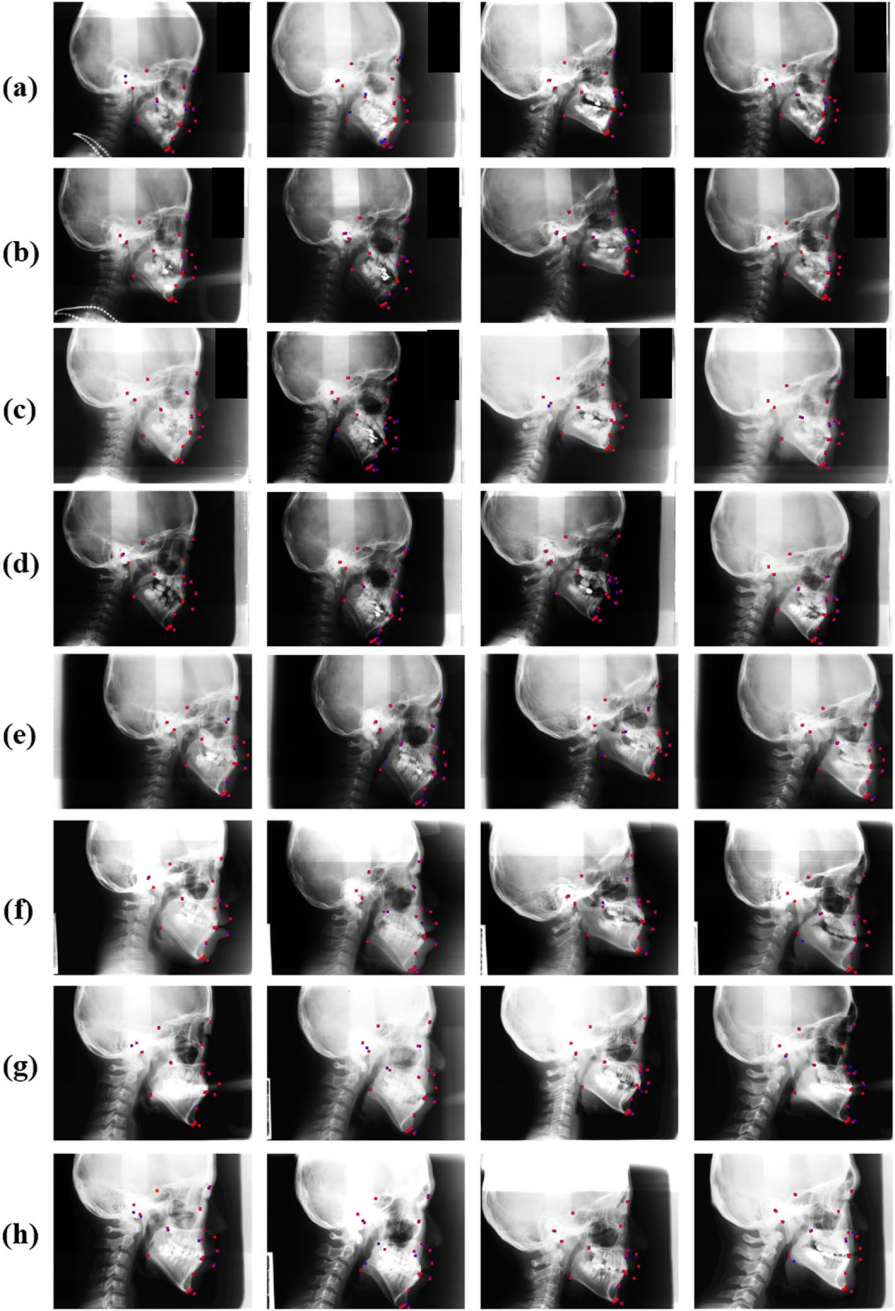
**a**-**h** Show representative detection results of cephalometric landmarks produced by Ceph-Net on the test dataset split by specific age (8 to 16 except for 12 years old). The red points denote the detected landmarks by detection networks, while the blue points present the ground truth of cephalometric landmarks

We also illustrated several representative examples of landmark detection results from Ceph-Net and popular detection networks. The results in Fig. 6 revealed that the proposed Ceph-Net detected cephalometric landmarks more accurately than the popular detection networks in challenging scanned lateral cephalograms such as cephalograms containing permanent dentition (Fig. 6a-c), mixed dentition (Fig. 6d-f), soft tissues with low contrast (Fig. 6b, d, and e), and hard tissues with low contrast (Fig. 6f). We compared the detection performance of cephalometric landmarks from different detection networks on specific conditions in scanned lateral cephalograms as shown in Fig. 7. The Ceph-Net also outperformed other detection networks on five specific conditions in scanned lateral cephalograms. Figure 8 shows the visual representative examples of landmark detection results produced by Ceph-Net on the test dataset split by each age (8–16 years old except for 12 years old).

From the ablation study in Table 3, Ceph-Net combined with the three modules not only showed performance improvement, but also when each module was integrated alone. The detection performance of cephalometric landmarks was improved from the MREs of $1.95\pm 2.97$ to $1.75\pm 1.67$ by simultaneously embedding modules in the Ceph-Net. Our method presented the best detection performance by combining the three modules and demonstrated the effectiveness of each module in the Ceph-Net.

**Table 3:** Ablation study results for each module in the Ceph-Net

Models			SDR (%)					MRE (mm)
SAM	CAM	MSI	1.0	2.0	3.0	4.0	5.0	
			33.61	68.15	83.01	90.18	94.17	1.95 ± 2.97
✓			38.65	71.63	84.34	90.61	94.35	1.81 ± 1.68
	✓		37.93	70.89	83.98	90.78	94.34	1.82 ± 1.67
		✓	36.36	69.31	82.78	89.57	93.59	1.89 ± 1.74
✓	✓		39.56	71.93	84.10	90.53	94.48	1.79 ± 1.68
✓	✓	✓	41.35	73.14	85.22	91.18	94.65	1.75 ± 1.67

Given the detected cephalometric landmarks, the scanned lateral cephalograms were classified into seven anatomical types in each clinical measurement. The main reason for detecting cephalometric landmarks in the orthodontic treatment field is the classification of anatomical types and the evaluation of dentofacial growth and development, diagnosis of skeletal and dental anomalies, treatment planning, and treatment outcome assessment. Seven clinical measurements including ANB, SNB, SNA, ODI, ADPI, FHI, and FMA were considered. In clinical measurements, a scanned lateral cephalogram can be categorized into three anatomical types using different geometrical criteria such as angle or distance between specific cephalometric landmarks. For the classification of the anatomical types, the geometrical criteria for the seven clinical measurements are described in Table 4. In Table 5, Ceph-Net obtained the best classification performance of approximately 76.42% compared with those from the other detection networks.

**Table 4:** Seven standard clinical measurements for anatomical type classifications

Measurements	Type 1 (Class 1)	Type 2 (Class 2)	Type 3 (Class 3)
ANB	$3.2^\circ -5.7^\circ$	$>5.7^\circ$	$<3.5^\circ$
SNB	$74.6^\circ -78.7^\circ$	$<74.6^\circ$	$<78.7^\circ$
SNA	$79.4^\circ -83.2^\circ$	$>83.5^\circ$	$<79.4^\circ$
ODI	$78.4^\circ -80.5^\circ$	$<80.5^\circ$	$<78.4^\circ$
APDI	$77.6^\circ -85.2^\circ$	$>77.6^\circ$	$>85.2^\circ$
FHI	$0.65-0.75$	$>0.75$	$<0.65$
FMA	$26.8^\circ -31.4^\circ$	$>31.4^\circ$	$<26.8^\circ$

**Table 5:** Quantitative comparison of classification accuracy for cephalometric analysis between Ceph-Net and other detection networks

Measurements	U-Net	SegNet	Dense U-Net	Attention U-Net	Ceph-Net
ANB	69.50	63.25	67.25	69.50	71.50
SNB	73.75	68.00	73.50	76.25	77.50
SNA	64.50	58.50	64.50	65.25	65.75
ODI	77.20	64.50	79.50	75.50	77.25
APDI	60.75	53.00	59.75	58.50	63.75
FHI	81.75	72.75	84.00	84.00	83.25
FMA	79.50	75.50	79.00	80.75	78.00
Mean	74.78	67.17	74.57	75.00	76.42

## Discussion

In orthodontics and maxillofacial surgery, cephalometric analysis is essential for accurate and reliable treatment planning and diagnosis. Cephalometric landmarks identify specific points on a scanned lateral cephalogram of the head, which is used as reference points for cephalometric analysis. The major challenges for cephalometric landmark detection are image quality and superimposed bilateral structures, which affect the reliability of landmark identification [15, 31]. The quality of an analog image is primarily decided during film exposure and the process of capturing and processing it, and there are limited options to enhance the image quality afterward [5]. Furthermore, when poor-quality analog films are scanned, the resulting images often appear even worse on screens, which can make it difficult to identify landmarks accurately and could potentially lead to more errors. [44].

Unlike digital lateral cephalograms, however, scanned lateral cephalograms have low image qualities with low contrast and resolutions, which causes inter- and intra-observer variability in cephalometric landmark identification [31]. Moreover, manual cephalometric analysis from each landmark is tedious and time-consuming. Therefore, automatic methods for the detection of cephalometric landmarks even in low-contrast and low-resolution scanned lateral cephalograms are required, which improves the overall accuracy and efficiency of cephalometric analysis. In this study, we proposed an attention-based stacked regression network (Ceph-Net) for automatic landmark detection on scanned lateral cephalograms with low contrast and resolutions. The main body of Ceph-Net was the stacked FCNs which progressively refined the detection of cephalometric landmarks on each FCN. By embedding DSAM and MCM in Ceph-Net, the network learned both local and global context and semantic relationships between cephalometric landmarks. Additionally, the deep supervision in each FCN further boosted the training stability and the detection performance of cephalometric landmarks.

We compared the detection performance of Ceph-Net with those of other popular detection networks such as U-Net, SegNet, Dense U-Net, and Attention U-Net. Ceph-Net achieved superior detection performance with lower MRE and higher SDR than the popular detection networks (Table 1). Our method could accurately detect cephalometric landmarks on scanned lateral cephalograms from children and adolescents with mixed and permanent dentitions between the ages of 8 and 16 years except for 12 years old (Fig. 6). Moreover, Ceph-Net demonstrated an accurate and consistent detection accuracy on the test dataset split by age from 8 to 16 except for 12 years old (Figs. 5 and 7). As shown in Fig. 6b, d, and e, the soft-tissue regions in the scanned lateral cephalograms have low contrast because soft tissues such as muscles, fat, and skin absorb X-rays to a lesser extent than the bones, teeth, and other hard tissues [45]. Compared Ceph-Net with other popular detection networks, Ceph-Net obtained the highest performance improvement in cephalometric landmarks (upper lip, lower lip, and subnasale) located in soft tissues (Fig. 4). Also, Ceph-Net outperformed the popular detection network in detecting nine cephalometric landmarks (sella, nasion, orbitale, porion, supramentale, pogonion, incision inferius, incision superius, and posterior nasal spine) located in hard tissues (Fig. 4). In Ceph-Net, the local and global context and semantic relationships between cephalometric landmarks on anatomical configuration were successfully learned in the proposed end-to-end learning manner, leading to accurate detection of cephalometric landmarks in low-contrast regions and morphological variations.

Ceph-Net outperformed other detection networks in the classification results of anatomical types (Table 5). Since the classification of anatomical types was measured by the angle and distance between specific cephalometric landmarks, the proposed DSAM captured long-range relationships between spatial and channel feature maps, which provided a positive effect on classification accuracy. Ceph-Net could perform automatic detection and analysis of cephalometric landmarks by learning semantic relationships between landmarks in scanned lateral cephalograms with low contrast and resolutions while reducing annotation time and analysis effort.

Compared with existing methods for cephalometric landmark detection [16, 17, 23–25], the Ceph-Net achieved comparable performance within the clinically acceptable accuracy range of 2.0 mm. All of the existing methods were performed using digital lateral cephalograms which had higher image quality than those of scanned lateral cephalograms. These disadvantages of scanned lateral cephalograms could lead to higher detection errors than digital lateral cephalograms [6]. Also, they built a dataset obtained from patients between the ages of 6 to 60 years, while we built our dataset from children and adolescents between the ages of 8 to 16 years. Different from fully grown adults, morphological variations in anatomy and growth among different children and adolescents led to significant variations in anatomical landmarks, including mixed dentition, permanent dentition, supernumerary teeth, and unerupted teeth [12]. Despite these challenges, the Ceph-Net showed superior detection performance within the clinically acceptable accuracy range of 2.0 mm even in specific conditions in scanned lateral cephalograms.

Some cephalometric landmarks such as the porion, gonion, posterior nasal spine, and articulare are more challenging than the other landmarks [46]. We also observed that the MRE of these cephalometric landmarks was higher than the other landmarks in Ceph-Net. This error is associated with the superimposition of craniofacial structures and the differential magnification of bilateral structures, as well as the low contrast and resolution of hard tissues in scanned lateral cephalograms [47]. The winding path of the ear canals generates multiple vertically overlapping radiolucent structures, which probably contributed to an identification error of porion [48]. The location of bilateral landmarks is defined as the midpoint of both sides, but it is difficult to estimate due to high inter- and intra-observer variability [49]. The imprecise superimposition of both jaws on the lateral cephalogram leads to errors in marking the gonion on either the left or right jaw [50, 51]. Also, this inherent property could bring about a negative effect on the detection performance [16].

The proposed method has several limitations. First, we only collected datasets of scanned lateral cephalograms from children and adolescents aged 8–16 years old to train detection networks. Therefore, when our method is extended to digital lateral cephalograms that are not used as training datasets, it is difficult to guarantee consistent detection performance of cephalometric landmarks. Second, Ceph-Net could have a potential limitation in generalizability when applied to external datasets because it was only evaluated using internal datasets. In future studies, we will improve the generalizability and clinical efficacy of Ceph-Net using large scanned and digital lateral cephalogram datasets acquired from both children and adults under various imaging conditions from multi-centers or devices. Further evaluation of linear distance measurements between cephalometric landmarks will be performed for applications in clinical practice such as analyzing the growth pattern. In addition, we plan to evaluate our methods using public datasets to ensure fairness and accuracy [31]. We expect this approach to be applied to detect anatomical landmarks on various poor-quality analog radiographs, beyond cephalometric radiographs.

## Conclusions

In this study, we proposed Ceph-Net for the automatic detection of cephalometric landmarks on scanned lateral cephalograms with low contrast and resolutions. Ceph-Net was designed to learn different semantics of anatomical structures among patients and long-range relationships between cephalometric landmarks by embedding our proposed modules in an end-to-end manner. The experimental results showed the Ceph-Net outperformed the popular detection networks for the detection and analysis of cephalometric landmarks. Therefore, Ceph-Net demonstrated the automatic detection and analysis of cephalometric landmarks by successfully learning local and global context and semantic relationships between cephalometric landmarks in scanned lateral cephalograms with low contrast and resolutions. Ceph-Net could provide clinicians with automatic cephalometric analysis in a scanned lateral cephalogram while reducing manual annotation time and analysis effort.

## Data Availability

The datasets generated and/or analyzed during the current study are not publicly available due to the restriction by the Institutional Review Board of Seoul National University, School of Dentistry in order to protect patients’ privacy but are available from the corresponding author on reasonable request. Please contact the corresponding author for any commercial implementation of our research.

## Abbreviations

FCN: Fully convolutional networks
ROIs: Regions of interests
2D: Two-dimensional
MSI: Multi-scale inputs
DSAM: Dual attention module
MCM: Multi-path convolution module
CEL: Cross-entropy loss
BN: Batch normalization
ReLU: Rectified linear unit
SAM: Spatial attention module
CAM: Channel attention module
FCEL: The CEL with deep supervision
MRE: Mean radial error
SDR: Successful detection rate
