# Correction: Ceph-Net: automatic detection of cephalometric landmarks on scanned lateral cephalograms from children and adolescents using an attention-based stacked regression network

**DOI:** 10.1186/s12903-023-03804-3

**Published:** 2024-02-01

**Authors:** Su Yang, Eun Sun Song, Eun Seung Lee, Se-Ryong Kang, Won-Jin Yi, Seung-Pyo Lee

**Affiliations:** 1 Department of Applied Bioengineering, Graduate School of Convergence Science and Technology, Seoul National University, Seoul, South Korea; 2 Department of Oral Anatomy, Dental Research Institute, School of Dentistry, Seoul National University, Seoul, South Korea; 3 Department of Biomedical Radiation Sciences, Graduate School of Convergence Science and Technology, Seoul National University, Seoul, South Korea; 4 Department of Oral and Maxillofacial Radiology and Dental Research Institute, School of Dentistry, Seoul National University, Seoul, South Korea

**Correction: BMC Oral Health 23, 803 (2023)**

**https://doi.org/10.1186/s12903-023-03452-7**

In this article [1], the dagger symbol has been inadvertently added for the corresponding authors.

The original article has been corrected.
